# Transforming Capillary Alginate Gel (Capgel) into New 3D-Printing Biomaterial Inks

**DOI:** 10.3390/gels8060376

**Published:** 2022-06-14

**Authors:** Andrew Philip Panarello, Corey Edward Seavey, Mona Doshi, Andrew K. Dickerson, Thomas J. Kean, Bradley Jay Willenberg

**Affiliations:** 1 Department of Internal Medicine, College of Medicine, University of Central Florida, Orlando, FL 32827, USA; panard@knights.ucf.edu (A.P.P.); corey.seavey@ucf.edu (C.E.S.); mona.mathew@ucf.edu (M.D.); 2 Department of Mechanical, Aerospace, and Biomedical Engineering, Tickle College of Engineering, University of Tennessee, Knoxville, TN 37996, USA; dickerson@utk.edu; 3 Biionix Cluster, Department of Internal Medicine, College of Medicine, University of Central Florida, Orlando, FL 32827, USA; thomas.kean@ucf.edu

**Keywords:** 3D printing, tissue engineering, alginate, gelatin, hydrogel, biomaterial ink, poly-L-lysine, polyelectrolyte complexation, scaffold, anisotropic, microgel

## Abstract

Three-dimensional (3D) printing has great potential for creating tissues and organs to meet shortfalls in transplant supply, and biomaterial inks are key components of many such approaches. There is a need for biomaterial inks that facilitate integration, infiltration, and vascularization of targeted 3D-printed structures. This study is therefore focused on creating new biomaterial inks from self-assembled capillary alginate gel (Capgel), which possesses a unique microstructure of uniform tubular channels with tunable diameters and densities. First, extrusions of Capgel through needles (0.1–0.8 mm inner diameter) were investigated. It was found that Capgel ink extrudes as slurries of fractured and entangled particles, each retaining capillary microstructures, and that extruded line widths *W* and particle sizes *A* were both functions of needle inner diameter *D*, specifically power-law relationships of *W*~*D*^0.42^ and *A*~*D*^1.52^, respectively. Next, various structures were successfully 3D-printed with Capgel ink, thus demonstrating that this biomaterial ink is stackable and self-supporting. To increase ink self-adherence, Capgel was coated with poly-L-lysine (PLL) to create a cationic “skin” prior to extrusion. It was hypothesized that, during extrusion of Capgel-PLL, the sheared particles fracture and thereby expose cryptic sites of negatively-charged biomaterial capable of forming new polyelectrolyte bonds with areas of the positively-charged PLL skin on neighboring entangled particles. This novel approach resulted in continuous, self-adherent extrusions that remained intact in solution. Human lung fibroblasts (HLFs) were then cultured on this ink to investigate biocompatibility. HLFs readily colonized Capgel-PLL ink and were strongly oriented by the capillary microstructures. This is the first description of successful 3D-printing with Capgel biomaterial ink as well as the first demonstration of the concept and formulation of a self-adherent Capgel-PLL biomaterial ink.

## 1. Introduction

The need for functional replacement tissues and organs is ever-growing [1]. The advent of tissue engineering and use of tissue scaffolds to create functional transplants in vitro is an innovative breakthrough for the medical community with great potential to address the shortfalls in donor/tissue organ supplies [2]. Three-dimensional (3D) printing and bioprinting have critically expanded tissue engineering approaches and made plausible the production of constructs that better replicate the complex structure and function of natural tissues and organs [3]. Such 3D printed/bioprinted tissues and organs could also potentially reduce the use of animal models in research by substitution with engineered tissues that closely mimic physiologies of interest with human rather than animal cells [4,5].

There are challenges, however, that are encountered with 3D printing in tissue engineering often due to the paucity of biomaterial inks that serve as the initial extracellular matrix (ECM) of printed tissue/organs, limited resolution of printers, and poor vascularization of the resulting 3D-constructs [6,7]. Hence, there is a need for new 3D printing biomaterial inks that are readily extruded (i.e., printable), self-supporting, and adhesive. Inks should possess appropriate, tunable bioactivity and porosity to facilitate robust colonization by target cells (i.e., biocompatible) [4,8,9,10,11]. Inspired by the pioneering work developing microgels as biomaterial inks by Burdick et al. [12,13,14], we hypothesized that capillary alginate gel (Capgel) tissue scaffolds are ideal candidate biomaterials than can be readily transformed to meet this need [15,16,17,18,19,20,21].

**Figure 1:**
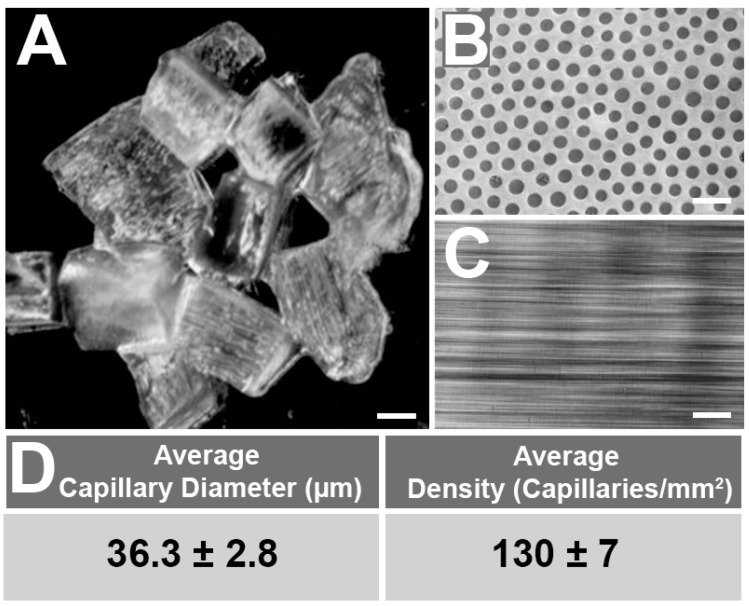
Capillary alginate hydrogel (Capgel) biomaterial tissue scaffolds have uniform microstructures of parallel, patent, regular tubular microchannels. (**A**) Gross stereomicrograph of cut Capgel pieces used to load syringe barrels. Phase-contrast micrographs of Capgel (**B**) imaged parallel to and (**C**) perpendicular to the capillary microstructure; (**D**) average capillary diameter and density of Capgel used in this study ± standard deviation (SD). Scale bar = 1 mm for (**A**) and 100 μm for (**B**,**C**).

Capgels are a unique family of self-assembled hydrogel biomaterials that have regular microarchitectures of cylindrical channels running in parallel throughout the bulk of the gel (Figure 1). The primary component of these hydrogels is alginate, a popular natural anionic linear polysaccharide biopolymer composed of β-D-mannuronic and α-L-guluronic acid residues [8,22]. Initial ionic crosslinking of an alginate solution via uniaxial diffusion of divalent metal ions such as Cu^2+^ generates the Capgel self-assembled microstructure (Section 4.1). Capillary diameter and density can be tailored for a given application via selection of the initial alginate and/or diffusing divalent metal ion (i.e., Cu^2+^) concentrations. Since alginate does not have cell attachment sites, gelatin, which has intrinsic Arg-Gly-Asp (RGD) cell-adhesion motifs [4,23,24], is added to the initial alginate solution [15]. After ionic crosslinking, these gels are sectioned into blocks, subjected to carbodiimide chemistry to form peptide crosslinks, undergo a series of washes, and then are terminally sterilized via autoclave to produce the final Capgel scaffolds (Section 4.2). These scaffolds were then cut into smaller Capgel pieces and loaded into a syringe for printing/extrusion (Section 4.3 and Section 4.4). Various Capgels that have been successful in a wide range of tissue engineering applications include: 3D stem cell culture scaffolds [19], injectable stem cell delivery [20], in vitro construction of functional nerve [17,18], and as injectable wound healing biomaterials [15,16].

In the present work, crosslinked Capgel pieces were extruded through various fine gauge needles to characterize extrusion width and particle size (area). Next, we assessed the printability, stackability, and self-supporting capacities of Capgel biomaterial ink through different 3D prints. A novel approach for increasing the self-adherence of Capgel ink through the formation of a polyelectrolyte complex “skin” on the outer surfaces of gel blocks with poly-L-lysine (PLL) prior to extrusion is detailed and tested. The biocompatibility of this new Capgel-PLL biomaterial ink is then evaluated through the culture of human lung fibroblasts (HLFs), a cell type known to support and facilitate vascularization [25,26,27,28], on/within the ink.

## 2. Results and Discussion

Capgel biomaterials formulated with 2% alginate/ 2.6% gelatin were successfully produced as previously described [15]. Capgel blocks cut into smaller pieces (Figure 1A) were used as the biomaterial ink for the extrusion and printing experiments. Microstructural characteristics (Figure 1B,C) of this Capgel are shown in Figure 1; capillary diameters and density were 36.3 ± 2.8 μm and 130 ± 7 capillaries/mm^2^ on average, respectively (Figure 1B,D). In the context of the present studies, it is likely most appropriate to consider Capgel as an elastomeric biomaterial ink, as it is a peptide-bond crosslinked network of alginate polysaccharide chains and gelatin polypeptide chains swollen in its presumed theta solvent water.

### 2.1. Capgel Ink Needle Extrusions

**Figure 2:**
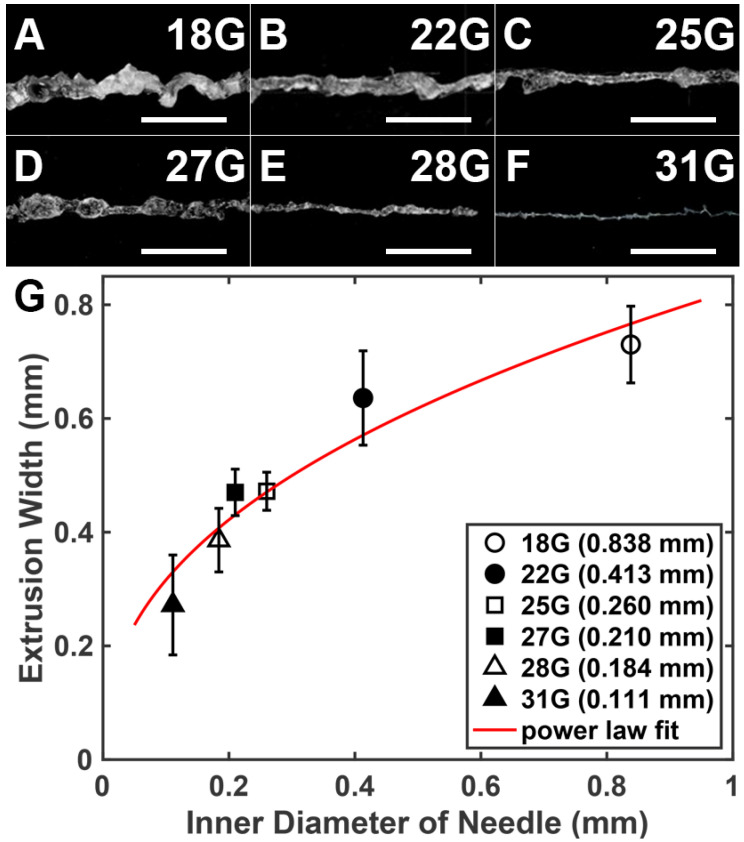
Widths of Capgel biomaterial ink extrusions from a range of different gauge needles follow a power-law relationship. (**A**–**F**) Stereomicrographs of Capgel needle extrusions; a needle gauge used for each is indicated in the upper right of each panel; (**G**) plot of average extruded line widths as a function of needle inner diameters (i.e., gauges). Scale bar = 5 mm for (**A**–**F**) and error bars = SD in (**G**).

Capgel ink extrusion characteristics were determined over a wide range of needle gauges (Figure 2). Analysis of optical micrographs of extruded lines of Capgel ink (Figure 2A–F) shows that extruded line widths decrease with increasing needle gauge as expected (Figure 2G). Furthermore, it was determined that the linkage between Capgel extruded line widths (*W*) and needle inner diameters (*D,* i.e., gauge) is well-described by a power-law scaling relationship. Specifically, fitting all individual data points, *W~D*^0.42^ (R^2^ = 0.80). The physics underlying the observed scaling are complex—Capgel is viscoelastic and is sheared and compressed through the extruder. Extruded line widths are also likely influenced by extrusion speed/volume and print X–Y translation speed. A more rigorous mathematical characterization of extrusion width with a needle diameter that accounts for Capgel properties and extruder/extrusion dynamics is beyond the scope of the present study and will be a focus of future work. This notwithstanding, Capgel was demonstrated as a biomaterial ink that exhibits predictable extrusion line width across a range of needle diameters, and these widths have practical importance regarding gross dimensions and resolution of 3D prints with Capgel ink.

**Figure 3:**
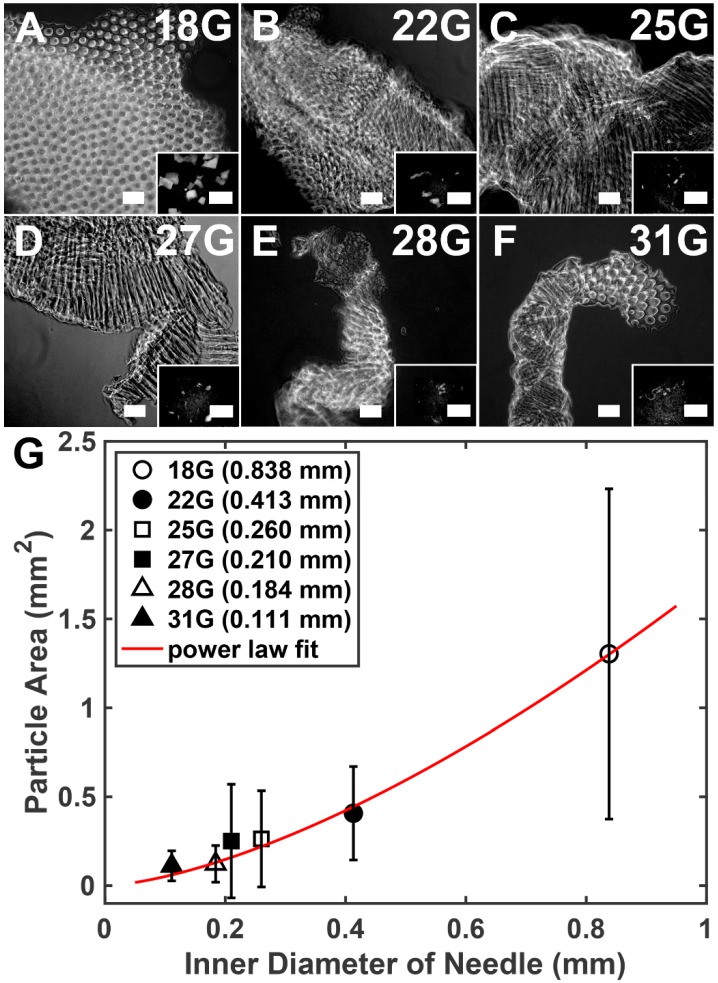
Sizes (areas) of sheared Capgel microparticles from extrusions using a range of different gauge needles follow a power-law relationship. (**A**–**F**) Phase-contrast micrographs of sheared Capgel microparticles formed during needle extrusions; the needle gauge used for each is indicated in the upper right of each panel. Insets show representative stereomicrographs of multiple sheared Capgel microparticles resulting from extrusion through each corresponding needle gauge; (**G**) plot of average microparticle areas (i.e., sizes) of extruded Capgel microparticles as a function of needle inner diameters (i.e, gauges). Scale bar = 100 μm for (**A**–**F**) and 1 mm for all insets; error bars = SD in (**G**).

Microgels are defined as hydrogel microparticles [14]. When Capgel blocks were forced through and extruded from fine gauge needles, lines of entangled microgels were formed due to compression and shearing (Figure 2A–F). Submerging these resultant lines of extruded Capgel ink in saline dispersed these microgels, enabling imaging and quantification of the microparticle sizes (Figure 3A–F; sizes described as the projected cross-sectional areas, mm^2^). These data show that average microgel size produced by the needle extrusions reduced dramatically over the 18 G to 31 G range (Figure 3G). Fitting all data points, the projected microparticle area *A* (i.e., microparticle size) and needle inner diameter also follows a power-law scaling relationship, specifically *A~D*^1.52^ (R^2^ = 0.49, Figure 3G). The value of the correlation coefficient is low because of the large variance in *A* produced by the largest needle; fitting only the average values of *A* yields R^2^ = 0.99. As shown in Figure 3, as the inner diameter of the needle decreases, the variation of produced microparticle sizes also decreases (Figure 3G), supporting the notion that higher gauge needles produce more consistent Capgel biomaterial ink extrusions. The exceptionally large standard deviation values for the 18-gauge needle may be due to the inherent elasticity of Capgel, and the strain and stress at fracture required to form microgel fragments via shearing through the needle. These data also suggest that the 22 G needle inner diameter and the corresponding shear forces applied to Capgel during extrusion may represent threshold values required to produce extrusions with consistent (i.e., less variable) properties. Extrusion characteristics and the coupling of these characteristics with needle gauge are also likely influenced by the size and shape of the Capgel pieces loaded into the syringe barrel for extrusion. It is also critically important to note that capillary microstructure was retained in all microgel particles produced by all extrusion conditions (Figure 3A–F), a vital property bolstering its use as a new 3D-printing biomaterial ink [14]. Capgel is viscoelastic and thus exhibits non-Newtonian and shear-thinning behavior. As such, the relation between shearing stress and rate of shearing strain is highly nonlinear. Furthermore, we posit that, as Capgel pieces are broken into smaller fragments by shear forces, the effective viscosity decreases. Thus, viscoelasticity is expected to be different for various needle gauges, prompting our investigation into the characteristics of Capgel extruded through 0.1–0.8 mm diameter needles.

### 2.2. 3D Printing with Capgel Ink

**Figure 4:**
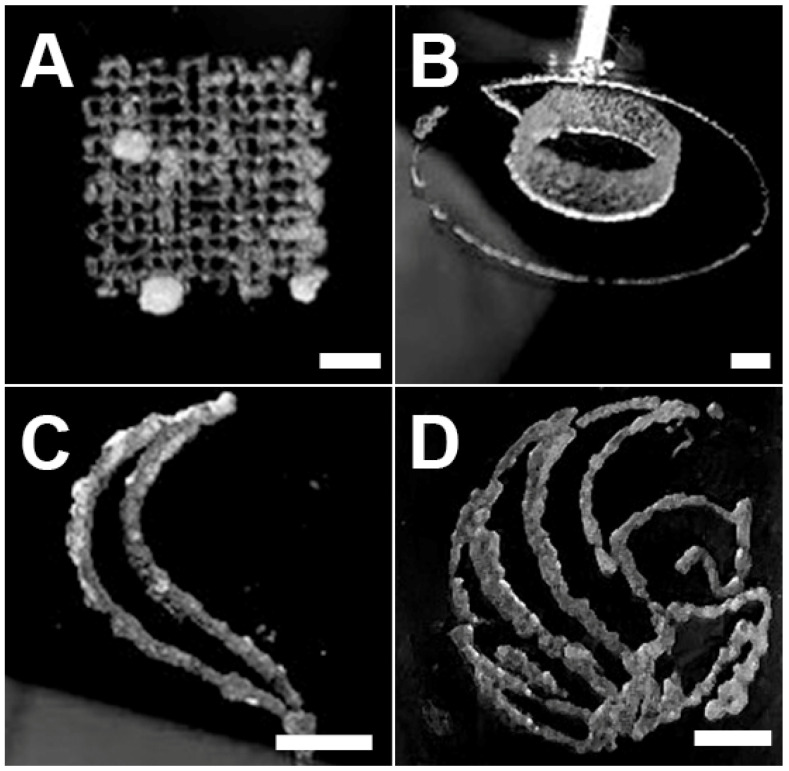
Capgel biomaterial ink can be used to print 3D structures of varying complexity. Images of (**A**) a 10 mm × 10 mm weave/mesh pattern; (**B**) a 10 mm-diameter cylindrical structure; (**C**) a single feather of the Pegasus graphic; and (**D**) a 10 mm-diameter Pegasus graphic. Scale bar = 2.5 mm for all.

A variety of structures were successfully 3D-printed with Capgel biomaterial ink including a weave/mesh pattern, an 18-layer cylindrical structure and Pegasus graphic (Figure 4). Based on the extrusion studies, all prints were executed with a 25 G tuberculin or 31 G insulin fused-needle syringe. The weave/mesh print was first attempted (Figure 4A). The success of this print demonstrates that Capgel ink can print single-extrusion width lines in close proximity with minimal bleed and discontinuities. Next, a layered cylindrical structure was attempted to assess the stackability and self-supporting capacities of Capgel biomaterial ink (Figure 4B, Video S1). The success of this 3D print demonstrates that the ink as printed has sufficient self-adhesion/cohesion to stack in successive layers [29] as well as support the weight of those layers. To investigate the potential of Capgel ink in a more elaborate 3D print with various curved and straight elements, a Pegasus graphic was produced. Once it was established that a single feather of the Pegasus could be printed (Figure 4C, Video S2), the full graphic was attempted with success (Figure 4D, Video S3).

It is evident that the greatest print fidelities were observed for the larger curved and circular elements present in the cylindrical and Pegasus prints. This potentially stems from tensile and compression forces at work when the 3D printer undertakes a dramatic change in direction, such as in the weave, and the interplay of those forces with the self-adhesivity/cohesivity and extrusion dynamics of the Capgel ink, which can result in print over-extrusions and/or discontinuities [30]. These experiments helped to inform adjustments to printing parameters such as extrusion and X–Y translation speed to improve fidelity of future prints, although additional optimizations will need to be made to increase print fidelity for patterns with short runs and sharp angles such as elements in the weave/mesh and Pegasus prints. Overall, these prints demonstrate that 3D-prints containing lines of various curvatures, a wide range of angles, and varying negative space elements can be achieved with Capgel biomaterial ink.

### 2.3. Development of Capgel-PLL Ink

**Figure 5:**
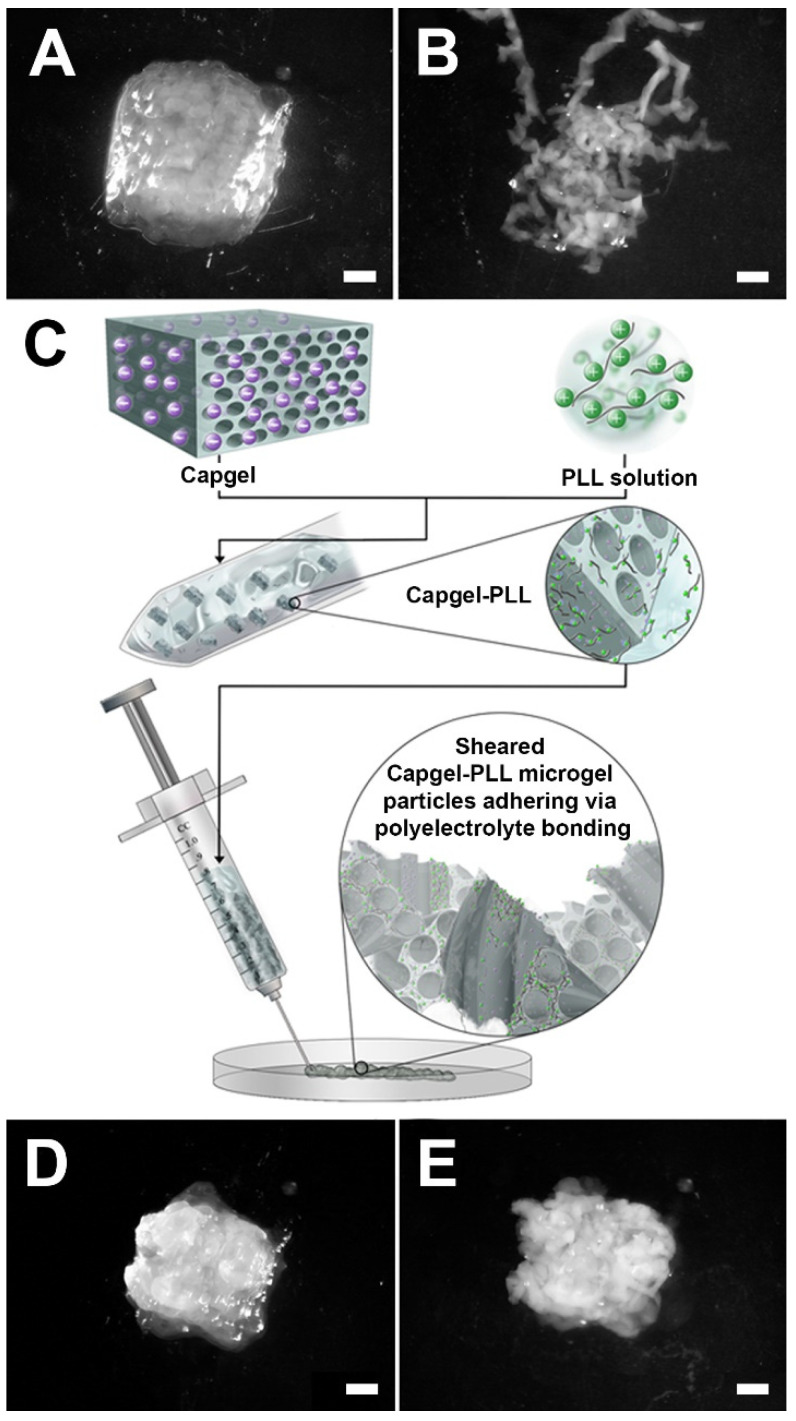
Capgel-PLL biomaterial ink self-adheres through shear-induced fragmentation followed by polyelectrolyte bonding. Capgel 25 G needle extrusions before (**A**) and after (**B**) submersion in saline; (**C**) illustration of the Shear-Induced Fragmentation Polyelectrolyte Bonding—SIFPeB—mechanism for Capgel-PLL self-adherence. Capgel-PLL 25 G needle extrusions before (**D**) and after (**E**) submersion in saline. Scale bar = 1 mm for all.

**Figure 6:**
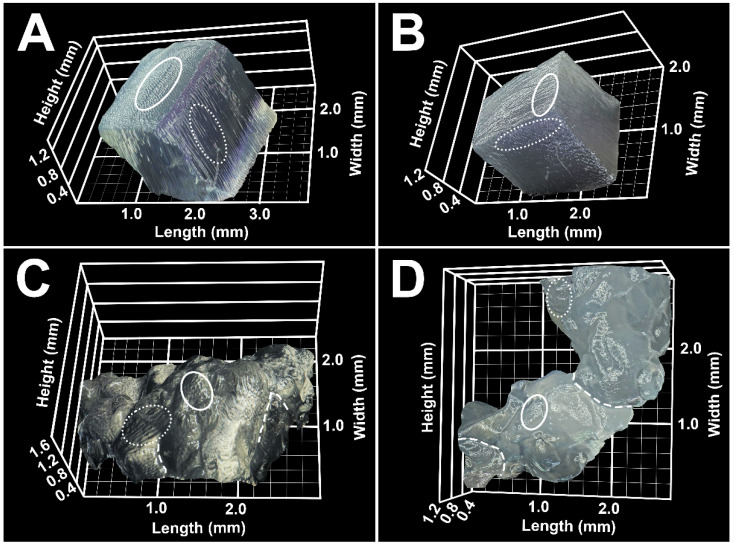
Microgels that comprise Capgel and Capgel-PLL needle extrusions retain the capillary microstructure present in each biomaterial prior to extrusion. Stereomicrograph 3D reconstructions of (**A**) Capgel and (**B**) Capgel-PLL cut pieces prior to extrusion. Representative areas of the capillary microstructures of each hydrogel piece are outlined with white ellipses; solid-line ellipses highlight the capillary microarchitecture viewed parallel to the capillary long-axis and dashed-line ellipses highlight capillary microarchitecture viewed perpendicular to this axis. Stereomicrograph 3D reconstructions of (**C**) Capgel and (**D**) Capgel-PLL 25 G needle extrusions; the different ellipses highlight the same orientations described above, and the white dashed lines highlight borders between entangled microgels.

The property of self-adherence is critical for biomaterial inks to sustain the 3D-printed shape in solution [6]. As shown in Figure 5, Capgel extrusions created with a fine gauge needle resulted in sheared slurries of entangled, fractured microparticles (Figure 5A) that tended to disperse when submerged in an aqueous fluid volume (Figure 5B) [15,16,21]. The long-range order of the capillary microstructure present in Capgel blocks (Figure 6A) is disrupted in these extrusions, but this microarchitecture is preserved in each entangled microgel particle (Figure 6C). To improve Capgel biomaterial ink cohesion when submerged in aqueous fluids, a polyelectrolyte-complex “skin” was first created on Capgel prior to printing by soaking blocks in dilute (0.05% *w*/*v*), sterile solutions of medium molecular weight (M_W_ = 30–70 kDa) positively-charged PLL. Polyelectrolyte complexation of alginate with PLL has been described previously [31,32], especially for the construction and application of alginate-PLL microspheres and microcapsules for drug and cell encapsulation and delivery [33,34,35,36] as well as for Capgel scaffold stabilization [19].

The novel theory to create self-adherent biomaterial inks with Capgel-PLL as the first example is that, as the PLL-coated blocks fragment while extruding through the needle, negatively-charged internal alginate carboxylic groups are revealed and can form polyelectrolyte bonds with the positively-charged PLL skin. This concept is captured in Figure 5C and was successful as shown by the improved cohesion evident for Capgel-PLL ink extrusions (Figure 5D,E). As with the Capgel extrusions, the long-range microstructural order of Capgel-PLL blocks (Figure 6B) was interrupted by the extrusion process, but, again, capillary microarchitectures were retained in each extruded microgel (Figure 6D). Compared to Capgel ink, the Capgel-PLL formulation required greater force to extrude, which was beyond the capabilities of the basic 3D-printer utilized in this study. Future 3D-printing studies with Capgel-PLL ink are therefore planned using a printer with a wider range of capabilities.

Printing and bioprinting with preformed biomaterials such as microgels and microspheres is a burgeoning area in biomaterial ink research. Examples include self-assembling microgels that are readily printable and stabilized through cell-to-microgel adhesive interactions during extrusion and UV-based crosslinking chemistry post-extrusion [12,13,14], and polyethylene glycol (PEG) hydrogel microsphere prints crosslinked with click chemistry [37]. The approach for biomaterial ink self-adhesion detailed here based on polyelectrolyte bonding formed as a result of biomaterial fragmentation caused by shearing during extrusion is novel and complements other previously described approaches [37].

### 2.4. Biocompatibility of Capgel-PLL Ink

**Figure 7:**
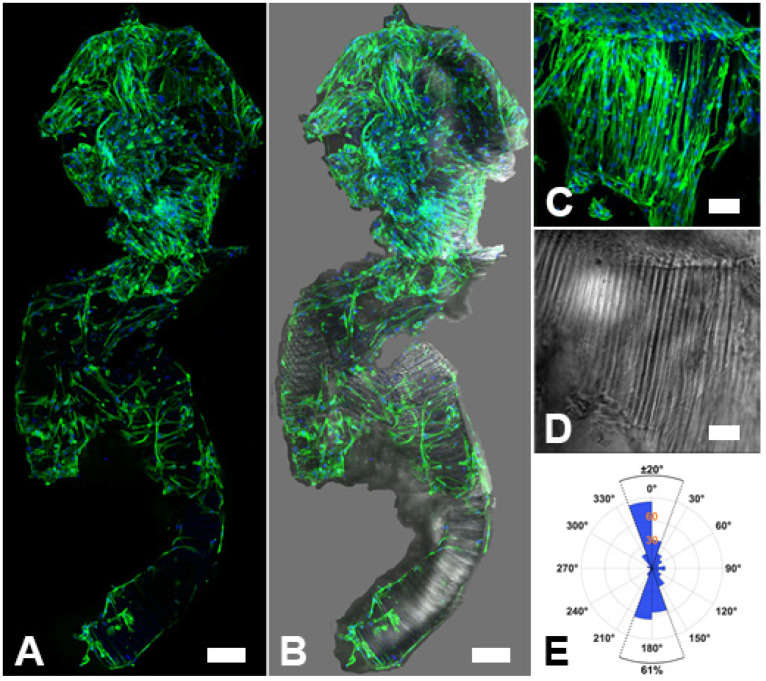
Human lung fibroblasts (HLFs) attach and spread on Capgel-PLL biomaterial ink extrusions in culture. (**A**) Large-area maximum z-projection confocal fluorescence mosaic micrograph composed of multiple contiguous image fields (10× mag) stitched together of HLFs colonizing a Capgel-PLL extrusion taken at one week in culture. (**B**) The same fluorescence micrograph as shown in (**A**) merged with the corresponding differential interference contrast (DIC) micrograph of the Capgel-PLL extrusion; (**C**) Maximum z-projection confocal fluorescence and (**D**) DIC micrographs of a microgel particle colonized by HLFs in another Capgel-PLL extrusion taken at one week in culture. (**E**) Polar plot of HLF nuclei orientations relative to the capillary long-axis from individual microgel particles in extruded Capgel-PLL like that shown in (**C**,**D**) colonized with cells that attached and spread during a one-week culture. Green fluorescence is from actin filaments stained with a conjugated phalloidin dye (Actingreen488) and blue fluorescence is from HLF nuclei stained with NucBlue. Scale bar = 200 μm for (**A**,**B**) and 100 μm for (**C**,**D**).

Evaluations of biocompatibility need to be undertaken for any new biomaterial formulation and/or application. Pursuant to this axiom, HLFs were seeded onto Capgel-PLL extrusions and cultured for a week. HLFs were selected as these cells are reported to support vascular formation and stability in co-culture with endothelial cells [7,25,26,27,28,29,38,39], which is the focus of ongoing studies. Following culture, cell-laden Capgel-PLL extrusions were fixed, treated with fluorescent dyes that stain actin filaments (phalloidin) and DNA (i.e., nuclei, DAPI) and imaged with laser-scanning confocal microscopy. Fluorescence and differential image contrast (DIC) images of these Capgel-PLL extrusions show that abundant HLFs had attached, spread, and colonized the Capgel-PLL biomaterial ink (Figure 7A–D). As noted above, the microgel particles produced during extrusion preserve capillary orientation within the microparticles themselves, but each were randomly oriented upon exit from the needle (Figure 7B,D). Furthermore, the cultured HLFs within the biomaterial ink were strongly oriented by the Capgel-PLL capillary microstructure (Figure 7C,D). Image analysis quantifying the nuclear orientation of cells colonizing these regions of the ink showed that 61% of the 397 nuclei imaged were aligned within ±20° with respect to the Capgel-PLL capillary long axis (Figure 7E). Taken together, these data strongly support the in vitro biocompatibility of Capgel-PLL biomaterial ink and its tissue scaffolding potential.

## 3. Conclusions

This is the first report of Capgel as a new 3D-printing biomaterial-ink. The extruded ink line widths and particle sizes were functions of needle gauge with both following power-law scaling relationships. Constructs 3D-printed with Capgel ink could support their own weight, could be complex, and each entangled microgel particle retained the self-assembled Capgel capillary microstructure. The produced desirable random arrangement of microgels with respect to each other in the prints could facilitate robust cell seeding, growth, colonization and tissue formation—including highly-branched microvascularization. This is also the first report of Capgel-PLL biomaterial ink, which has increased self-adherence achieved through a novel mechanism: Shear-Induced Fragmentation Polyelectrolyte Bonding (SIFPeB). Capgel-PLL ink was also found to be biocompatible, supporting the survival, attachment and spreading of cultured HLFs as well as inducing cell orientation via the ink capillary microstructure. This work represents the start of an innovative new avenue for future 3D-printing and 3D-bioprinting investigations.

## 4. Materials and Methods

### 4.1. Formation and Growth of Capgel Hydrogels

Capgel hydrogels were synthesized as previously described [20]. In short, 10% bloom gelatin (G1890, Sigma-Aldrich, Saint Louis, MO, USA) was prepared in distilled, deionized water (ddH_2_O) and degraded by heating with sodium hydroxide (NaOH) at 80 °C for 72 h. Oligomeric 10% gelatin solution was equilibrated to ambient temperature (–23 °C) for 2 h. Sodium alginate powder (Protanal Pharm Grade LF10/60, FMC Biopolymer, Philadelphia, PA, USA supplied by IMCD US) was then added to create a final solution of 2% alginate and 2.6% gelatin in ddH_2_O.

Capgel parent gel was grown in a 10 cm glass Petri dish coated with dehydrated 4% alginate. The parent solution was added to the alginate coated Petri dish, which was placed within a larger glass dish. The parent solution was covered with a 0.5 M copper (II) sulfate (CuSO_4_, Acros Organics, Flanders, Belgium) soaked Kimwipe, which was held in place with a plastic ring and 0.5 M CuSO_4_ was dripped over portions of the Kimwipe directly covering the growing parent gel. The Kimwipe was removed and the parent gel and Petri dish were completely submerged in 0.5 M CuSO_4_. After 72 h, the Capgel was cut into strips, which were then rinsed 3× in ddH_2_O each day for four days in a covered plastic container. After ddH_2_O washes, the strips were sectioned into ~5 × 5 × 3 mm blocks.

### 4.2. Crosslinking and Preparation of Capgel Blocks

Capgel blocks were crosslinked via carbodiimide chemistry as previously described [20]. Per 50 mL conical tube, four (4) ~5 mm × 5 mm × 3 mm Capgel blocks were cross-linked via carbodiimide chemistry in 20 mL PBS containing 1.89 mg/mL *N*-Hydroxysuccinimide (NHS) (ThermoFisher, Waltham, MA, USA) which was allowed to soak in the gel overnight at 4 °C followed by adding 20 mL PBS with 1.57 mg/mL *N*-(3-Dimethyl-aminopropyl)-*N′*-ethylcarbodiimide hydrochloride EDC (Sigma-Aldrich, Saint Louis, MO, USA) to yield a final reaction volume of 40 mL. The cross-linking reaction was then gently shaken overnight at 4 °C. After cross-linking, Capgel blocks were washed 3× with 0.2 μm filter- sterilized (564-0020, Nalgene, NY, USA) 0.9% NaCl (S271-3, ThermoFisher, Waltham, MA, USA) in ddH_2_O (sterile saline) over three days in a covered plastic container, followed by 3 washes with 0.2 μm filter-sterilized 10× sodium citrate solution (BP1325-4, ThermoFisher, Waltham, MA, USA) over four days. Finally, the blocks were washed 3× in sterile saline over four days. Capgel in saline was then autoclaved with a liquid cycle and sterilization hold of 15 min. Sterilized Capgel blocks were then stored at 4 °C in sealed glass bottles.

### 4.3. Capgel Capillary, Particle Size, and Extrusion Analysis

Average capillary size and density of used Capgel were found using NIH ImageJ software (v1.52b, National Institutes of Health, Bethesda, MD, USA). A sample size of 25 capillaries per gel was used to determine the average gel diameter of three separate samples. Using the circle tool in ImageJ, Capgel capillaries were outlined and their diameters measured. These diameters were then averaged and the standard deviation calculated.

Diced pieces of Capgel were loaded into the barrels of syringes with a range of different gauge needles using a sterile spatula (Corning Inc., Corning, NY, USA) to determine the average size of the microgel particles produced by extruding the gel through the needle. The gauges (G) tested were 31 G, 28 G, 27 G, 25 G, 22 G, and 18 G. Syringe needles 18 G, 22 G, 25 G, and 27 G (Becton Dickinson, Franklin Lakes, NJ, USA) were attached to a 1 mL Luer-lok syringe (Becton Dickinson, Franklin Lakes, NJ, USA). The 28 G and 31 G needles were fused to a 0.5 mL fixed head tuberculin syringe (Becton Dickinson, Franklin Lakes, NJ, USA). Sufficient amounts of Capgel were added to each syringe to fill ¼ to ½ of the total volume of the syringe. The gel was then extruded from each syringe onto a plastic Petri dish (Corning Inc., Corning, NY, USA) and were imaged using a stereo microscope and the images processed and analyzed using ImageJ. To determine extruded line widths, five points were selected in 3 mm intervals along the length of each extruded line and their thicknesses measured. For extruded microgel particle sizing, the method described by Ramalingam et al. was followed to capture the microgel particle area using ImageJ [40]. Averages and standard deviations for extruded lines widths and microgel particle areas were calculated and power-law relations were determined with MATLAB (vR2021b, MathWorks, Natick, MA, USA).

### 4.4. 3D Printer Settings and Methods

In a biosafety cabinet, a Seed R3bel bioprinter was used to print the Capgel fragments through a blunt ended 25 G or 31 G needle. Stereolithography (STL) files were sliced (Slic3r v1.3.0, slic3r.org), with settings of 3 mm filament diameter, an extrusion multiplier of 4X and a print speed of 30 mm/s, to create a gcode file for printer control. Pronterface software (Printrun v1.6.0, pronterface.com) was used to control the desired print(s) on the bioprinter: an extrusion rate adjustment of 300% and a print speed adjustment of 15% were then used. The structures were printed onto sterile 10 cm plastic Petri dishes (Corning, NY, USA) and covered until it was time for imaging.

Four models were tested for printing with Capgel material. The first was a ~1 cm × 1 cm “weave” with a rectilinear infill pattern that was 2 layers high. The second model tested was a hollow cylinder with a 1 cm diameter, consisting of eighteen layers of height, which resulted in a height of –3 mm. The third and fourth models came from the same Pegasus source file. The third model was a “feather” from the Pegasus, while the fourth model was a 1 cm diameter print of the entire Pegasus model consisting of one extrusion layer.

### 4.5. Poly-L-Lysine Coating of Capgel (Capgel-PLL)

Capgel was coated with poly-L-lysine (PLL) to create a polyelectrolyte complex (PEC) “skin” on the hydrogel (Capgel-PLL). Briefly, in a biosafety cabinet, sterilized Capgel blocks were transferred onto a sterile Petri dish. A sterile scalpel was then used to dice the blocks into ~2 mm × 2 mm × 2 mm Capgel pieces. Sterile solutions of 0.05% (*w*/*v*), 30–70 kDa PLL; Sigma-Aldrich, MO, USA) were made, and filter sterilized (Nalgene, NY, USA). Once sterilized, 10 mL of PLL solution were added to a 15 mL conical tube together with diced Capgel pieces made from five (5) blocks and allowed to rotate on an orbital shaker (Orbitron Rotator II, Boekel, PA, USA), overnight, at room temperature. The Capgel pieces were then rinsed with three 30 min washes of 40 mL sterilized ddH_2_O on an orbital shaker. Depending on the experiment, the pieces were submerged in saline or DMEM cell culture media (Gibco, Waltham, MA, USA) and allowed to soak at 37 °C overnight. Then, Capgel-PLL pieces were loaded into 25 G tuberculin syringes (26046, EXELint, Redondo Beach, CA, USA) and extruded onto 10 cm plastic Petri dishes for imaging using a stereo microscope with a digital camera (MU1803, AmScope, Irvine, CA, USA), or extruded into culture plates for cell studies.

### 4.6. Digital 3D Reconstruction of Capgel and Capgel-PLL Pieces and Extrusions

Optical micrographs and 3D reconstructions of Capgel and Capgel-PLL pieces and extrusions were acquired and generated using the Keyence VHX 7100 digital microscope with the Keyence VHX 7000 software (Keyence Corporation of America, Itasca, IL, USA). All images were acquired with E20 lens and ×80 digital zoom. The tilting angles of the lens with respect to the sample were 22° (Capgel piece), 23° (Capgel extrusion), −23° (Capgel-PLL piece) and −12° (Capgel-PLL extrusion). Samples were illuminated with a full ring light during imaging.

### 4.7. Cultivation and Preparation of HLF Cells

Normal human lung fibroblasts (NHLF CC-2512, Lonza, Basel, Switzerland) were cultured at 37 °C in T-75 culture flasks using Dulbecco’s Modified Eagle Medium (DMEM) with 10% fetal bovine serum (FBS; Thermofisher, Waltham, MA, USA), 1% Glutamax, and 1% penicillin/streptomycin (Gibco, Waltham, MA, USA).

### 4.8. Post-Extrusion Cell Seeding of Extruded Capgel-PLL, Histological Staining, and Image Processing

Extruded Capgel-0.05% PLL structures were seeded with normal HLFs (NHLF CC-2512, Lonza, Basel, Switzerland). This was accomplished by trypsinization (0.05%, Gibco, Waltham, MA, USA) of the cells, centrifugation at 1912 RCF (Centra GP8R, 216 4-place swinging bucket rotor, Thermofisher, Waltham, MA, USA), and addition of 160,000 HLF cells over the entire extruded structure. The cells were then allowed to attach to the cell media-soaked print. After 2 h, 2 mL of media were added to the Petri dish, and the print was cultured at 37 °C for 1 week, changing media every 2 days. After 1 week, cellularized-Capgel was washed 3× in phosphate buffered saline (PBS; Sigma-Aldrich, Saint Louis, MO, USA) before fixation with 4% paraformaldehyde for 30 min at room temperature. Following fixation, cellularized-Capgel was rinsed 3× with PBS and then permeabilized with 0.2% Triton-X 100 for 30 min at room temperature. After 3 washes with PBS, cellularized-Capgel was stained with NucBlue Live and ActinGreen 488 ReadyProbes (Invitrogen; Carlsbad, CA, USA) for 2 h in darkness. Finally, 3 rinses with PBS were performed prior to imaging on FluoroDish (FD35-100, World Precision Instruments Inc., Sarasota, FL, USA). Imaging was performed using the Zeiss 710 laser scanning confocal microscope at 10× magnification with excitation wavelengths of 405 nm and 488 nm for NucBlue Live and ActinGreen 488, respectively. Images were processed with Zen 2010 software (Zeiss; Oberkochen, Jena, Germany).

Z-stacks of each channel (DIC, NucBlue, and actin-green) were overlaid and converted into maximal z-projections with ImageJ version 1.52b (National Institutes of Health; Bethesda, MD, USA). Images were stitched together using Adobe Photoshop (2022 v23.3.2, Adobe Inc., San Jose, CA, USA) and image background removed with a magnetic lasso tool. To determine orientation of HLF cell nuclei, measures were made of cell-colonized microgels that were, by chance, oriented with the capillary long-axis parallel to the imaging plane, which was readily determined by visual inspection of the corresponding DIC images. NucBlue channel images of these areas were rotated such that the long-axis of the capillaries was vertical. Images were then binarized by applying thresholding in ImageJ, and subsequently processed with the ‘region props’ function in MATLAB. Nuclei were approximated as elliptical blobs with major and minor axes. Nuclear orientation is that of the major axis with respect to the image vertical axis. Those nuclei with centroids above the horizontal midline of each image have possible orientation values of −90°–90°, with those below the midline having orientations 90°–270°. Extrusions, HLF culture, and nuclear orientation measurements were only carried out on Capgel-PLL, not on gels without capillary microstructures.

## Data Availability

All data for this study are available from the corresponding author upon request.

## Supplementary Materials

The following supporting information can be downloaded at: https://www.mdpi.com/article/10.3390/gels8060376/s1, Video S1: Cylinder 5× playback; Video S2: Feather 4× playback; Video S3: Pegasus 4× playback.
